# Impact of the Interaction between Aquatic Humic Substances and Algal Organic Matter on the Fouling of a Ceramic Microfiltration Membrane

**DOI:** 10.3390/membranes8010007

**Published:** 2018-02-01

**Authors:** Xiaolei Zhang, Linhua Fan, Felicity A. Roddick

**Affiliations:** 1 Chemical and Environmental Engineering Department, School of Engineering, RMIT University, GPO Box 2476, Melbourne, VIC 3001, Australia; linhua.fan@rmit.edu.au (L.F.); felicity.roddick@rmit.edu.au (F.A.R.); 2 Department of Environmental Engineering, Kyungpook National University, 80 Daehak-ro, Buk-gu, Daegu 702-701, Korea

**Keywords:** humic acid, fulvic acid, algal organic matter, microfiltration, ceramic membrane, fouling

## Abstract

The influence of the interaction between aquatic humic substances and the algal organic matter (AOM) derived from *Microcystis aeruginosa* on the fouling of a ceramic microfiltration (MF) membrane was studied. AOM alone resulted in a significantly greater flux decline compared with Suwannee River humic acid (HA), and fulvic acid (FA). The mixture of AOM with HA and FA exhibited a similar flux pattern as the AOM alone in the single-cycle filtration tests, indicating the flux decline may be predominantly controlled by the AOM in the early filtration cycles. The mixtures resulted in a marked increase in irreversible fouling resistance compared with all individual feed solutions. An increase in zeta potential was observed for the mixtures (becoming more negatively charged), which was in accordance with the increased reversible fouling resistance resulting from enhanced electrostatic repulsion between the organic compounds and the negatively-charged ceramic membrane. Dynamic light scattering (DLS) and size exclusion chromatography analyses showed an apparent increase in molecular size for the AOM-humics mixtures, and some UV-absorbing molecules in the humics appeared to participate in the formation of larger aggregates with the AOM, which led to greater extent of pore plugging and hence resulted in higher irreversible fouling resistance.

## 1. Introduction

Ceramic membranes are used increasingly in water and wastewater treatment due to their inherent advantages over conventional polymeric membranes such as narrow and well-defined pore size distribution, high surface hydrophilicity, and good mechanical and chemical stability [1]. Although the capital cost for the application of ceramic membranes is still higher than polymeric membranes, the longer lifespan and the ability of ceramic membranes to pair with a wider range of pre-treatment approaches have made them an effective alternative technology to compensate for the higher cost [2]. However, membrane fouling due to the presence of naturally-occurring aquatic organic matter in source waters is a major challenge in the application/operation of both conventional polymeric and ceramic membranes [3,4]. The fouling is generally related to the formation of a gel/cake layer by colloidal/particulate organic matter on the membrane surface, and adsorption/entrapment of small organic molecules within the membrane pore structure [5,6]. The characteristics of the organic matter in feed water are regarded as one of the critical factors affecting the fouling process [7].

Aquatic natural organic matter (NOM) is ubiquitous in natural water bodies, and can be classified as being of autochthonous and allochthonous origin [8]. Humic substances, such as humic (HA) and fulvic acids (FA), are one of the major fractions in allochthonous NOM, and it has been demonstrated in several studies that they can cause both reversible and irreversible fouling of microfiltration and ultrafiltration membranes in drinking water treatment [9,10]. As an autochthonous source of NOM, the organic matter released from microalgae during algal blooms in water storages can negatively impact the water treatment processes [8]. A number of studies have shown that algal organic matter (AOM) can cause severe fouling of both polymeric and ceramic membranes [11,12,13,14,15,16,17,18]. In these studies, the high molecular weight biopolymer-like compounds (such as polysaccharides and proteins) were identified as the key foulants causing a severe reduction in membrane efficiency [19,20]. 

Some recent studies showed that the effect of molecular interaction between humic substances and biopolymer-like substances (such as polysaccharides and proteins) could contribute to the fouling of low-pressure polymeric membranes. Xiao et al. [21] investigated the fouling characteristics of a polymeric UF membrane using HA, bovine serum albumin (BSA), and sodium alginate (SA) as model compounds. They found that the order of total fouling resistance for the various mixtures of the compounds was HA+SA > BSA+SA > HA+BSA, which was attributed to their different molecular weight and surface charge distributions resulting from interactions between these compounds. Myat et al. [22] also investigated the impact of the possible interactions between HA, BSA, and SA on the fouling of a polypropylene MF membrane. They found alginates or BSA (as model biopolymer compounds) formed large aggregates with humic acid, which could negatively affect the MF performance. However, the above studies have focused on polymeric membranes, which are significantly different from ceramic membranes in terms of physical, chemical, and mechanical properties. Moreover, the origin of the commercial humic acids used in those studies was not well defined, but were probably derived from soil, coal, or peat, which might not be the best representatives of aquatic humic substances [23]. To date, there is no published information regarding the effect of the interaction between AOM, aquatic NOM and its humic fractions (i.e., HA and FA) derived from natural surface waters on the fouling of ceramic water treatment membranes. 

The aim of this study was to investigate the influence of the interaction between the AOM released from *Microcystis aeruginosa* (the most common bloom-forming cyanobacteria in natural waters) and the humics in well-characterized Suwannee River organic matter (i.e., HA, FA) on the fouling of a commercially available ceramic MF membrane. This would allow an enhanced understanding of the impact of the co-occurrence of the humics and AOM in feedwater on the fouling of ceramic membrane systems and, hence, development of strategies to control the fouling. The interaction between the organic substances was examined in terms of the changes in molecular size, molecular weight, surface charge, and hydrophilicity.

## 2. Materials and Methods

### 2.1. Preparation of MF Feed Solutions

A synthetic water containing AOM and Suwannee River humics in MilliQ water was used as MF feedwater. The AOM was derived from *M. aeruginosa* (CS 566/01-A01) purchased from the CSIRO Microalgae Research Centre (Tasmania, Australia). The algal cultures were grown in 5 L Schott bottles at 22 °C using MLA medium [24] under humidified aeration. A 16/8 h light/dark cycle was used to simulate natural light conditions. Algal cultures were harvested at the 35th day of growth (stationary phase). Centrifugation (3270× *g* for 30 min) of the algal cell suspensions and subsequent filtration of the supernatant with 1 µm membranes (Whatman^®^, Grade GF/A, Maidstone, UK) were conducted to extract the soluble AOM. 

Suwannee River HA and FA were obtained from the International Humic Substances Society (USA). The stock solutions (50 mg DOC L^‒1^) were prepared by dissolving the organic matter into Milli-Q water, and the stock solutions were filtered using 1 µm membranes (Whatman^®^, Grade GF/A, Maidstone, UK) to remove any non-dissolved substances. The stock solutions were further diluted with deionized water to prepare the MF feed solutions containing single and mixed compounds, respectively, for examining their individual and combined fouling effect. The composition of the feed solutions is given in Table 1 (the error for DOC was ~0.05 mg L^−1^).

According to the isolation protocols for Suwannee River HA and FA [25] and NOM [26], the HA and FA represent the high molecular weight and low molecular weight fraction of the humic substances, respectively. The mixture of HA and FA was used to resemble the hydrophobic fraction of the Suwannee River NOM. The AOM concentration was fixed at 2 mg DOC L^‒1^ in order to mimic a real algal bloom situation [27]. The pH of all feed solutions was regulated to 7 by using 1 mM NaOH or HCl. The ionic strength of the feed solutions was adjusted to 1 mM with NaCl prior to each run.

### 2.2. Microfiltration Tests 

A seven-channel tubular ceramic membrane with a nominal pore size of 0.1 µm (CeRAM^TM^ INSIDE, TAMI Industries, Nyons, France) was used in the MF experiments, which were operated under dead-end mode. The ceramic membrane surface layer was made of ZrO_2_ and the support layer was made of TiO_2_. The membrane surface was considered as highly hydrophilic as ZrO_2_-based membranes usually have a contact angle less than 20° due to the presence of surface hydroxyl groups [28]. All filtration runs were carried out at a constant transmembrane pressure (TMP) of 70 ± 1 kPa at room temperature (22 ± 2 °C). Approximately 2 L of each feed solution was filtered and permeate flux was recorded continuously. After each MF test, the membrane was backwashed for 2 min with deionized water. The same membrane was used for all MF runs, and after each run the membrane was restored by cleaning in place using 0.05 M NaOH and 0.05 M HNO_3_ solutions until the permeate flux reached 200–220 LMH. 

The fouling resistance (*R*) values can be calculated by Equations (1) and (2) using the flux (*J*) values determined before filtration, at the end of the filtration and after backwash. The *R_total_* refers to the total fouling resistance after MF of the AOM solutions. The *R_reversible_*/*R_irreversible_* is associated with the reversible/irreversible fouling resistance and *R_membrane_* is the clean membrane resistance.

(1) $$J=\frac{\Delta P}{\mu R_{total}}$$

(2) $$R_{total}=R_{membrane}+R_{reversible}+R_{irreversilbe}$$

### 2.3. Analytical Methods

DOC and UV absorbance at 254 nm (UVA_254_) were determined using a Sievers 820 TOC analyzer and a UV-VIS spectrophotometer (UV2, Unicam), respectively. pH was measured with a Hach Sension 156 pH meter. 

The hydrodynamic radius of the organic compounds was determined by using the dynamic light scattering technique with an ALV-5200 F spectrometer equipped with a compact goniometer. The sample was illuminated with a He-Ne laser of wavelength 632.8 nm and the intensity was measured at a scattering angle of 90°.

The zeta (ζ) potential values of all the feed water samples were determined using a Malvern Zetasizer Nano ZS (Malvern Instruments, Malvern, UK). The ζ potential was calculated based on the Henry equation using the Smoluchowski model. The electric field was applied to the clear disposable folded capillary zeta cell (DTS1070) for the measurement. Three measurements for each trial were carried out with the average values reported. 

The apparent molecular weight distribution of the AOM was determined by size exclusion chromatography (SEC) with LC-OCD at the Water Research Centre of the University of New South Wales, Australia. The LC-OCD system (LC-OCD Model 8, DOC-Labor Dr. Huber, Karlsruhe, Germany) utilized a SEC column (Toyopearl TSK HW-50S, diameter 2 cm, length 25 cm). The analyzer is equipped with an organic carbon detector (OCD) and a UV detector (UVD, responds to UV-absorbing compounds at 254 nm), and the chromatograms were processed using the Labview-based program Fiffikus (DOC-Labor Dr. Huber, Karlsruhe, Germany). The details of this technique are described by Huber et al. [29].

Non-ionic macroporous resins (DAX-8 and XAD-4) were employed to separate the organics into hydrophobic (HPO), transphilic (TPI), and hydrophilic (HPI) fractions. More details of the organic matter fractionation procedure can be found from Aiken et al. [30]. All filtration tests were duplicated and analyses triplicated, and results are reported in terms of mean value and error/standard deviation. 

## 3. Results and Discussion

### 3.1. Microfiltration of the Solutions Containing Individual and Mixed Compounds

**Figure 1:**
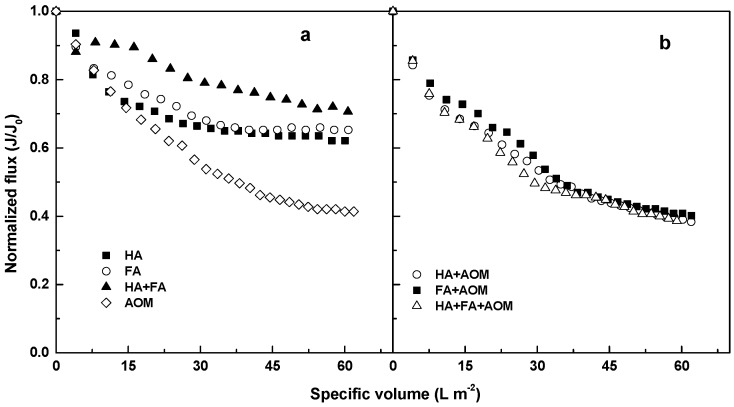
Flux profiles for the MF of the solutions containing (**a**) AOM (DOC = 2 mg L^−1^), HA (DOC = 2 mg L^−1^), FA (DOC = 2 mg L^−1^) and HA+FA (DOC = 2 mg L^−1^), respectively; (**b**) HA+AOM (DOC = 4 mg L^−1^), FA+AOM (DOC = 4 mg L^−1^), and HA+FA+AOM (DOC = 4 mg L^−1^), respectively.

The normalized flux for the MF of the solutions containing AOM, HA, FA, and NOM individually and their mixtures is shown in Figure 1. AOM alone gave a significantly greater flux decline compared with the other organic compounds, with approximately 60% of flux decline obtained at the end of the single cycle filtration (Figure 1a). The humic acid (HA) resulted in only slightly greater flux decline compared with the fulvic acid (FA) (i.e., 34% cf. 30%). The mixture of HA and FA exhibited less flux decline compared with the other compounds, with 24% flux reduction obtained at the end of the filtration. 

The presence of AOM in the HA, FA, and HA+FA solutions led to a much greater flux decline at the specific permeate volume of 60 L m^−2^ compared with the solutions containing only humics (Figure 1b). However, the solutions of mixed compounds gave a very similar flux decline. This indicates the flux performance of the ceramic MF membrane in the single-cycle filtration of the organic mixtures was predominantly governed by the AOM. 

**Figure 2:**
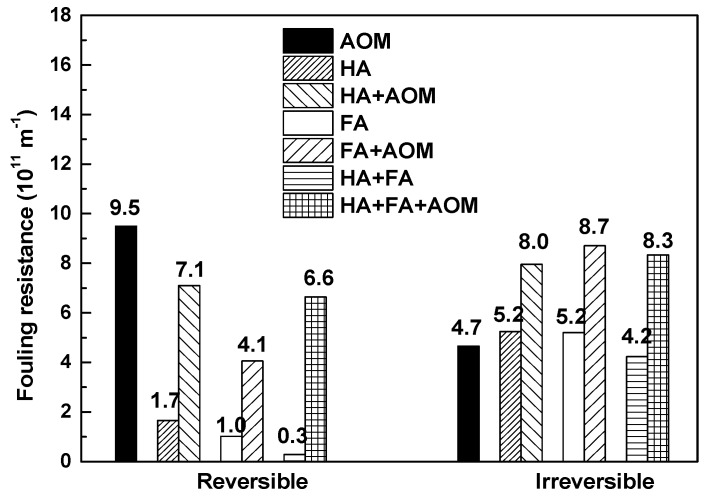
Comparison of membrane fouling resistance resulting from the various feed solutions.

The fouling resistance resulting from the various MF feeds is presented in Figure 2. The solution containing AOM led to the highest reversible fouling resistance, but lower irreversible fouling resistance compared with the other solutions except for HA+FA. HA+FA resulted in slightly higher values for reversible and irreversible fouling resistance these resistances than those values for HA or FA alone. HA alone resulted in slightly higher reversible fouling resistance compared with FA, but similar irreversible fouling resistance.

Addition of humics to the solutions containing AOM resulted in a markedly increased irreversible fouling resistance, which was approximately two-fold greater than for AOM alone. This also led to a significant increase in reversible fouling compared with the solutions containing humics only as a result of doubled DOC content of the feed, although the resultant reversible fouling resistance was lower than that for the solution containing AOM only. 

### 3.2. Characterization of the Feed Solutions

#### 3.2.1. Hydrodynamic Molecular Size

**Figure 3:**
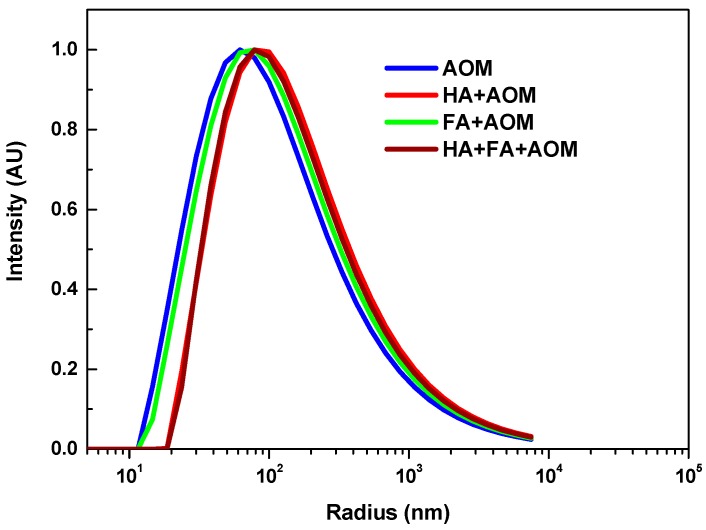
Comparison of the average hydrodynamic radius of AOM, HA+AOM, FA+AOM, and HA+FA+AOM.

The molecular size distributions for the AOM and humics+AOM solutions were examined using dynamic light scattering. The distribution of the hydrodynamic radius for the organic compounds covered a wide size range which was probably due to their polydispersed nature (Figure 3). However, a slight shift of the peaks towards larger radius was observed after mixing the AOM with HA, FA, or HA+FA, which was attributed to the physicochemical interactions (such as complexation and charge neutralization) between AOM and the organics in these solutions [31]. The combination of HA+FA+AOM, and HA+AOM gave higher average molecular size compared with those of FA+AOM and AOM alone.

#### 3.2.2. Solution Zeta Potential

The zeta potential of the AOM and humics+AOM solutions was measured to examine the surface charge of the individual and mixed organic compounds, and hence provide further information about the interaction between the compounds (Table 2). HA had a larger negative ζ potential than HA+FA and AOM, while the FA gave the smallest negative ζ potential. Mixing AOM with HA, FA, and HA+FA resulted in more negatively-charged ζ potentials compared to AOM. The HA+AOM, FA+AOM and HA+FA+AOM mixtures had considerably large negative ζ potentials. This suggested that the enlarged organic matter formed by humics-AOM interaction was moderately stable [32], which meant they had a fairly low potential for further self-aggregation. 

#### 3.2.3. Molecular Weight Distribution

**Figure 4:**
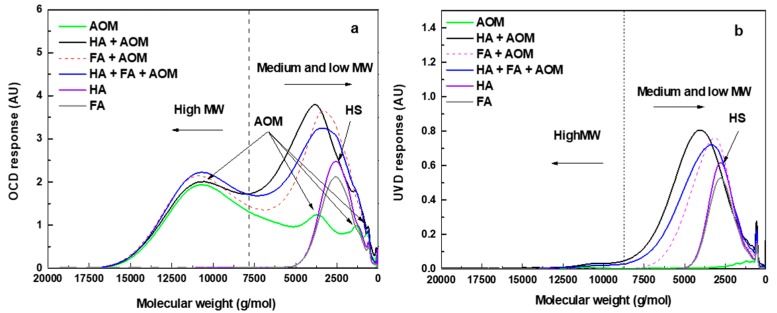
LC-OCD-UVD diagram for AOM, HA and FA (DOC = 2 mg L^−1^), HA+AOM (DOC = 4 mg L^−1^), FA+AOM (DOC = 4 mg L^−1^), and HA+FA+AOM (DOC = 4 mg L^−1^), (**a**) OCD response; and (**b**) UVD response (HS = humic substances).

The apparent molecular weight distributions of the AOM, HA, FA, and humics+AOM mixtures were examined using SEC with LC-OCD-UVD (Figure 4).

For the organic carbon detector (OCD) response (Figure 4a), the HA and FA showed pronounced peaks at a molecular weight (MW) of around 2500 g/mol. The AOM contained four major peaks at the MWs of approximately 11,000, 4000, 1200, and 750 g/mol. The high MW substance peaks of AOM (1st and 2nd peaks) were associated with high MW biopolymers and some biopolymers with lower MW (such as low MW polysaccharides, polypeptides and polyamino acids) compared with the first biopolymer peak [33]. 

All mixtures of AOM with the humic substances exhibited strong peaks at MW of around 11,000 and 2500–5000 g/mol. The second peak for the AOM-humic mixtures was much higher than that for the AOM and humics alone.

When UV detector (UVD) response was used (Figure 4b), AOM alone showed only one small peak at the MW of around 11,000 g/mol. Similar to the OCD response, HA and FA had very large peaks at MW around 2500 g/mol. Pronounced peaks for HA+AOM, FA+AOM and HA+FA+AOM occurred at the MW between 2500 and 5000 g/mol, and very small high MW peaks could be seen for HA+AOM and HA+FA+AOM.

#### 3.2.4. Fractionation of Organics in the Feed Solution

Resin fractionation showed that, based on DOC, over 50% of the AOM was hydrophilic, and the HPO and TPI fractions accounted for 28% and 21%, respectively (Table 3). The majority of the organic matter in the HA, FA, and HA+FA solutions was hydrophobic, with less than 20% of it being hydrophilic. 

Fractionation of the AOM-humics mixtures showed that the HPO fraction accounted for more than 50% of the DOC for all these solutions (Table 4). These mixtures contained a similar amount of TPI (0.4–0.6 mg DOC L^−1^), which accounted for less than 15% of the total DOC of each. The FA+AOM and HA+FA+AOM solutions contained a greater amount of HPO, but a smaller amount of HPI compared with the HA+AOM solution.

### 3.3. Membrane Fouling Mechanism

Addition of humic substances to the feed solution containing AOM led to the formation of large AOM-humics aggregates/complexes and the solution became more negatively-charged than that for the AOM solution. These changes affected the performance of the ceramic MF membrane as shown by the filtration tests in which the mixtures of AOM and aquatic humics resulted in a marked increase in hydraulically-irreversible fouling, although the initial flux decline pattern was fairly comparable for the AOM and AOM+humics solutions. 

It is well known that size exclusion is the core mechanism for low pressure membrane filtration processes [6,17], where the organic matter with larger molecular size in the feed water can normally lead to higher reversible fouling resistance during the filtration process. In addition to size exclusion, other fouling mechanisms (such as pore plugging and electrostatic adsorption) may also occur simultaneously during the MF process [6]. The higher reversible fouling and lower irreversible fouling resistance caused by the HA+AOM solution compared with FA+AOM and HA+FA+AOM was attributed to its larger molecular size and more negative ζ potential, where the more negative solution charge could prevent the molecules from adhering to the negatively-charged membrane [17]. The HA+FA+AOM solution had slightly lower molecular size but lower negative ζ potential than the HA+AOM solution, which resulted in its slightly lower reversible fouling and higher irreversible fouling than for the HA+AOM solution. Similarly, the lower reversible fouling for FA+AOM than for the other two mixtures was due to its lower molecular size and less negative ζ potential. These results suggest that the electrostatic interactions between the solution compounds and ceramic membrane were an important factor in causing reversible/irreversible fouling.

It is seemingly contradictory to the above claim that the MF of AOM alone gave the highest reversible fouling resistance, despite its having the lowest mean molecular size and less negative ζ potential. A possible explanation is that the high MW biopolymer-like compounds (such as transparent exopolymer particles (TEP)) in AOM tend to self-aggregate and, hence, form a larger and thicker cake layer on the membrane surface. This type of organics was reported to be fairly sticky and had fairly large impact on the fouling of low pressure polymeric UF membrane [34]. For the AOM-humics mixtures, the surface characteristics of the high MW biopolymer-like compounds were altered due to the interactions between the AOM and humics, which resulted in the lower reversible fouling caused by AOM-humics mixtures compared with AOM alone. In addition to the organic molecular size and ζ potential, the hydrophilicity of the solution also affected the MF performance where higher amounts of HPO compounds were associated with higher irreversible fouling, as reported by Qu et al. [17]. As shown in Table 3 and Table 4, AOM had significantly lower amounts of HPO compounds than the other solutions, which was in accordance with the lowest irreversible fouling contributed by AOM. 

According to a review of the literature, some studies reported that the humic material could encapsulate the biopolymer-like compounds (such as polysaccharides and proteins), forming larger compounds [22,35,36]. Such interaction could be revealed by the changes in their molecular weight distributions by using LC-OCD-UVD according to Myat et al. [22] who found the additional peaks for a BSA-humic acid mixture appeared at higher MW position (shorter retention time) than the BSA peak due to the BSA-humic acid interaction. In Figure 4a,b HA and FA showed strong OCD response peaks only at a MW of around 2500 g/mol, whereas all the other AOM-humics solutions displayed strong peaks at MW greater than 3000 g/mol. This indicated that the molecular size of the medium MW humic-like compounds in HA and FA was increased to some extent in the presence of AOM. Such peak shifting was also observed in Figure 4b, which indicated that the UV-absorbing material in HA and FA participated in forming higher MW substances in the presence of AOM. Besides, no obvious peaks at the position indicating a MW greater than 2000 g/mol in the LC-UVD diagram for AOM could be found, indicating that the UV-absorbing organics in AOM did not participate in forming the AOM-humics complexes.

Furthermore, there was very little difference in the size of the biopolymer peaks for AOM and the mixed solutions. This indicates that very high MW biopolymer compounds (larger than 8000 g/mol) were unlikely formed as a result of the mixing of the AOM and the humics. According to our previous studies, the organic compounds smaller than 10,000 g/mol could significantly contribute to hydraulically irreversible fouling [20]. This also explains why MF of AOM-humic solution caused higher irreversible fouling resistance compared with the AOM alone, as the mixtures contained large amounts of these compounds as a result of the AOM-humic interaction.

## 4. Conclusions

Although the flux decline pattern for the AOM derived from *M. aeruginosa* and its mixtures with humic substance was comparable in the single-cycle filtration tests, the mixtures of AOM and humic substances resulted in a reduction in reversible membrane fouling and a marked increase in irreversible fouling compared with AOM alone. 

The addition of aquatic humics to the solutions containing AOM led to a small increase in average molecular radius and molecular weight compared with AOM alone, due to the interaction between AOM and the humics. It is suggested that the UV-absorbing materials in the humics can bond with the AOM molecules to form higher MW/larger molecules leading to increased irreversible fouling resistance. 

The AOM-humic mixtures exhibited a more negative ζ potential than the individual compounds which was related to the higher UVA_254_ rejection and higher reversible fouling of the membrane. This indicates that the electrostatic interactions between the organic compounds, and between the organic matter and the membrane, would contribute considerably in forming reversible and irreversible fouling of the ceramic membrane. 

This study demonstrated that the presence of both AOM and humics in the influent of the ceramic membrane filtration systems for drinking water treatment can result in more severe irreversible membrane fouling, and hence the need for higher frequency of backwash and chemical cleaning of the membranes. The long term effect of the interaction between AOM and aquatic humics on the ceramic membrane systems should be investigated through further lab multi-cycle filtration and/or pilot studies. This would be beneficial for membrane plant operators to implement effective measures to control fouling during cyanobacterial blooms in their water storages. 

## Figures and Tables

**Table 1:** Feed water composition.

Solution	Composition (mg DOC L^−1^)
HA	2
FA	2
HA+FA	1 + 1
AOM	2
HA+AOM	2 + 2
FA+AOM	2 + 2
HA+FA+AOM	1 + 1 + 2

**Table 2:** Summary of the ζ potential for the feed solutions.

	Average ζ Potential
AOM	−27
HA	−43
FA	−19
HA+FA	−30
HA+AOM	−44
FA+AOM	−33
HA+FA+AOM	−39

**Table 3:** The fractional components of humic subtances and AOM.

	HA	FA	HA+FA	AOM
mg DOC L^−1^				
HPO	1.6 ± 0.1	1.5 ± 0.2	1.5 ± 0.2	0.6 ± 0.1
TPI	ND	0.07 ± 0.03	0.04 ± 0.02	0.4 ± 0.1
HPI	0.4 ± 0.1	0.4 ± 0.1	0.4 ± 0.1	1.0 ± 0.1

ND = not detected.

**Table 4:** The fractional components of humic-AOM mixtures.

	HA+AOM	FA+AOM	HA+FA+AOM
mg DOC L^−1^			
HPO	2.0 ± 0.2	2.7 ± 0.1	2.9 ± 0.2
TPI	0.5 ± 0.1	0.6 ± 0.1	0.4 ± 0.1
HPI	1.5 ± 0.3	0.6 ± 0.1	0.6 ± 0.2
